# A Novel Supported Polymer Inclusion Membrane Concept for Reagent-Efficient Membrane Design

**DOI:** 10.3390/membranes16040135

**Published:** 2026-04-01

**Authors:** Nasim Khatir, Enriqueta Anticó, Clàudia Fontàs

**Affiliations:** Chemistry Department, University of Girona, C/Maria Aurelia Capmany 69, 17003 Girona, Spain; nasim.khatir@udg.edu (N.K.); enriqueta.antico@udg.edu (E.A.)

**Keywords:** polymer inclusion membranes, supported membranes, cellulose filter paper, Durapore^®^ PVDF membranes, Aliquat 336, arsenate transport, reagent-efficient membrane fabrication

## Abstract

This work explores, for the first time, a novel strategy for the preparation of polymer inclusion membranes (PIMs) based on their deposition onto porous supporting substrates, introducing the concept of supported PIMs as a reagent-efficient alternative to conventional free-standing membranes. The approach aims to improve the sustainability of PIM fabrication by significantly reducing the amount of polymer and extractant required while preserving membrane functionality. PIMs were prepared using the two most widely employed base polymers, cellulose triacetate (CTA) and poly(vinyl chloride) (PVC), with Aliquat 336 as extractant. The total reagent consumption was reduced to half of the conventional formulation for CTA-based membranes and to one quarter for PVC-based membranes. Two porous supports with contrasting physicochemical properties—a hydrophilic cellulose filter paper and a hydrophobic Durapore^®^ PVDF membrane—were investigated. The supported membranes were characterized by contact angle measurements, SEM, FTIR, and TGA, confirming the successful integration of the PIM phase onto the porous supports without chemical alteration. Arsenate (As(V)) transport, preconcentration, and membrane reusability were evaluated. CTA-based supported PIMs exhibited transport efficiencies of approximately 90–95%, comparable to free-standing PIMs, whereas PVC-based systems showed a stronger dependence on membrane loading. Notably, CTA-based Durapore^®^–PIMs retained around 70% transport efficiency after three reuse cycles. These results demonstrate the feasibility of supported PIMs as a strategy for reducing membrane material consumption while preserving functional performance.

## 1. Introduction

Polymer inclusion membranes (PIMs) have attracted considerable attention as efficient and selective platforms for the extraction and separation of a wide range of metallic and non-metallic species. Their straightforward fabrication, fast transport kinetics, and ability to couple extraction and back-extraction within a single membrane phase distinguish them from conventional solvent-based techniques, enabling reduced energy consumption and minimal use of hazardous organic solvents [1,2]. A typical PIM consists of an extractant immobilized within the polymeric matrix of a base polymer, most commonly cellulose triacetate (CTA) or poly(vinyl chloride) (PVC), both of which exhibit excellent compatibility with widely used carriers such as Aliquat 336 (trioctylmethylammonium chloride) [1]. Owing to its dual role as extractant and plasticizer, Aliquat 336 often eliminates the need for additional additives, contributing to simpler membrane formulations and enhanced operational versatility [2,3].

In parallel with improvements in selectivity and transport efficiency, increasing attention has been directed toward enhancing the sustainability of membrane fabrication. Conventional PIM preparation typically involves relatively high amounts of polymer and extractant to ensure sufficient mechanical integrity and transport performance. Although effective, such formulations increase material consumption and may limit the overall sustainability of the process. Recent advances in PIM research have largely focused on the development of novel carriers, polymer functionalisation strategies and high-performance polymer matrices, expanding their applicability in environmental technologies while promoting more sustainable separation approaches [4]. For example, Sellami et al. developed CTA/poly(butylene adipate-co-terephthalate) blend membranes containing Aliquat 336 for the removal of Cr(VI) to improve mechanical stability, compatibility, and transport selectivity, while maintaining homogeneous membrane morphology [5]. Similarly, Bahrami et al. investigated PVDF-HFP-based (poly(vinylidenefluoride-co-hexafluoropropylene)) PIMs incorporating Aliquat 336 or alternative ionic liquid carriers, demonstrating that membrane flexibility, compatibility and extraction performance could be enhanced through careful optimization of polymer–carrier–plasticizer ratios [6]. However, comparatively less attention has been paid to strategies aimed at reducing the total quantity of membrane-forming reagents without compromising functionality.

In related areas of membrane and materials science, structural design and composite architectures have been explored to enhance performance and functional integration [7,8]. Although these approaches were not specifically developed for PIM systems, they highlight the potential of structural design as a complementary strategy to enhance membrane efficiency.

Building on this perspective, the present work explores the concept of supported PIMs, in which the active membrane phase is deposited onto a porous substrate to provide mechanical stability while minimizing reagent use.

To evaluate the influence of substrate properties on membrane formation and transport behaviour, two commercially available supports with contrasting physicochemical characteristics were selected: a hydrophilic cellulose filter paper and a hydrophobic poly(vinylidene fluoride) (PVDF) membrane.

Cellulosic materials have gained particular attention in recent years due to their low cost, widespread availability, and favourable physicochemical properties [9]. Among them, cellulose—commonly found in filter paper—has emerged as an attractive platform for eco-friendly analytical and separation devices, often serving as a porous scaffold for functional materials [10]. Its intrinsic hydrophilicity, high surface area, porous architecture, flexibility and mechanical robustness make cellulose-based paper especially suitable for sustainable sorptive and separation applications [11]. Previous studies have demonstrated that cellulose-based substrates can be functionalized to act as active platforms for metal ion sensing, adsorption, and separation. Paper-based analytical devices have been developed through the immobilization of selective reagents onto cellulose fibres, transforming the substrate from a passive support into an active separation or sensing medium [11]. In a related approach, Jayawardane et al. reported the integration of a PIM into a paper-based sensor, where cellulose filter paper served as the hosting platform for the membrane phase, enabling analyte transport and signal generation [12]. Furthermore, chemical modification of cellulose filter paper has been employed to enhance metal ion rejection and adsorption performance in filtration systems, highlighting the versatility of cellulose matrices in separation processes [13]. These studies underline the potential of cellulose as a structurally robust and chemically adaptable porous scaffold for membrane-based applications.

PVDF, in contrast, represents a hydrophobic porous material widely used in membrane-based separation technologies due to its excellent mechanical strength, chemical resistance, and defined pore structure. Beyond its use as a support material, PVDF and its copolymer PVDF-HFP have also been extensively employed as base polymers in PIM fabrication [14,15,16,17]. Numerous studies have confirmed the suitability of PVDF-based PIMs for metal ion extraction and separation, including systems incorporating Aliquat 336 or related quaternary ammonium carriers for selective transport of various metal species [6,18,19,20,21]. In addition, commercially available PVDF membranes, such as the hydrophobic Durapore^®^ membrane, provide high permeability and operational stability, making them attractive candidates as porous supports in membrane configurations [22].

Arsenic (As) was selected as the target analyte due to its well-established toxicity and the stringent regulatory limit of 10 µg L^−1^ set by the World Health Organization (WHO) for drinking water. PIMs have been widely investigated for arsenic removal using different membrane compositions and carriers [23,24]. For example, Govindappa et al. reported a recycled PVC-based PIM containing benzalkonium chloride as a carrier, achieving significant As removal over 24 h [25]. Zawierucha et al. demonstrated efficient arsenate (As(V)) transport using a CTA-based PIM incorporating Cyanex 921, reaching high transport and separation efficiencies from acid mine drainage solutions [26].

Among the various extractants employed, Aliquat 336 has proven effective for the transport of As(V), owing to its ability to exchange anionic species present at pH values above ~2. Under neutral conditions, As(V) exists predominantly as H_2_AsO_4_^−^ and HAsO_4_^2−^, which can be efficiently transported through CTA- and PVC-based PIMs containing Aliquat 336. In contrast, arsenite (As(III)) remains largely untransported due to its neutral speciation [27]. Building on this behaviour, Aliquat 336-based PIMs have been applied in preconcentration devices for low-cost As(V) detection in groundwater [28]. Comparative studies have further demonstrated that both CTA and PVC provide suitable matrices for As(V) transport, typically with optimal Aliquat 336 contents in the range of 40–50% (*w*/*w*) [24,29,30].

Despite the extensive body of work devoted to material and compositional modifications in PIM systems, comparatively little attention has been paid to structural approaches aimed at reducing membrane mass through the use of porous supports. In particular, the influence of support-assisted architectures under reduced membrane loading has not been systematically investigated.

The present study addresses this gap by introducing a supported PIM concept based on the deposition of conventional CTA- and PVC-based formulations onto porous substrates with contrasting physicochemical properties. By integrating reduced reagent consumption with a comparative analysis of polymer matrices and support materials, this work establishes supported PIMs as a viable and adaptable strategy for more sustainable arsenic separation.

## 2. Materials and Methods

### 2.1. Reagents and Solutions

The polymers CTA (Acros Organics, Geel, Belgium) and PVC (Sigma-Aldrich, Zwijndrecht, The Netherlands) and the extractant Aliquat 336 (Sigma-Aldrich, St. Louis, MO, USA) were used to prepare the PIMs. The reagents were dissolved in chloroform (Merck, Darmstads, Germany) in the case of CTA and tetrahydrofuran (THF) (PanReac-AppliChem, Barcelona, Spain) in the case of PVC. The supports were the common cellulose laboratory filter paper (size: 420 × 520 mm) (Filtros Anoia, Barcelona, Spain), and Durapore^®^ PVDF membrane (GVHP, 0.22 µm pore size; Merck Millipore, Dublin, Ireland).

As(V) stock solution (1000 mg L^−1^) in ultrapure water was prepared by dissolving the corresponding amount of the solid Na_2_HAsO_4_.7H_2_O (Merck, Darmstads, Germany). The stripping solution (0.1 M NaCl) was prepared using NaCl (PanReac-AppliChem, Barcelona, Spain). Arsenic ICP standard (1000 mg L^−1^ As) (Sigma-Aldrich, Buchs, Switzerland) was used to prepare standard solutions.

All reagents and solvents were of analytical grade, and the ultrapure water used to prepare the aqueous solutions was obtained using the Milli-Q Plus water purification system (Millipore Iberica S.A., Barcelona, Spain).

### 2.2. PIM Preparation

Supported PIMs were prepared using a conventional solvent casting approach, adapted to allow deposition of the PIM phase onto porous substrates. Polymeric solutions were prepared with a polymer-to-extractant mass ratio of 50:50, using 0.1 g polymer for a 9 cm diameter Petri dish. Therefore, for all supported configurations, the total PIM mass was 0.2 g (0.1 g polymer and 0.1 g Aliquat 336). In comparison, conventional free-standing PIMs typically employ higher polymer loadings, such as 0.2 g for CTA-based membranes [27,28,29] and 0.4 g for PVC-based membranes [24,29], while maintaining the same polymer-to-extractant ratio. Accordingly, the total amount of membrane-forming material was reduced by approximately 50% for CTA-based systems and 75% for PVC-based systems.

Circular discs of cellulose filter paper (thickness 115 ± 3 µm) or Durapore^®^ PVDF membranes (thickness 117 ± 2 µm) were placed at the bottom of the Petri dish, and the polymeric solution was cast directly onto the supports to ensure uniform surface coverage.

**Figure 1:**
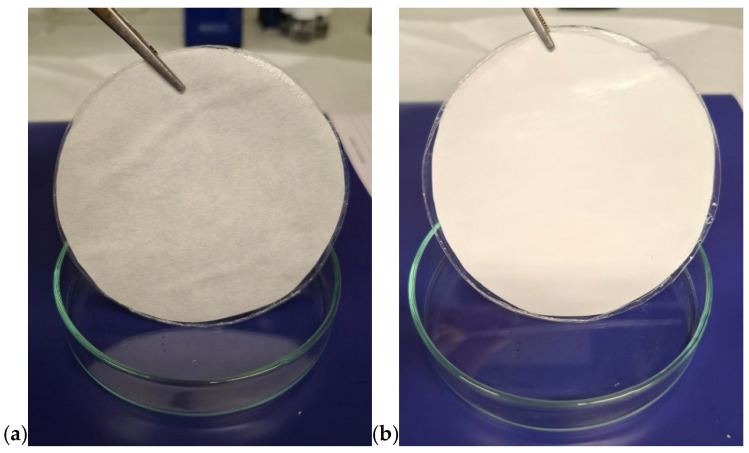
Supported PIMs: (**a**) filter paper–PIM; (**b**) Durapore^®^–PIM.

After solvent evaporation, the supported membranes—denoted as filter paper–PIM and Durapore^®^–PIM (Figure 1)—were obtained.

The resulting supported membranes exhibited overall thicknesses of 128 ± 1 µm (CTA) and 119± 2 µm (PVC) when prepared on filter paper, and 120 ± 2 µm (CTA) and 121 ± 1 µm (PVC) when deposited on Durapore^®^ supports.

### 2.3. PIM Characterization

The contact angles of the membranes’ surfaces were measured using a commercial contact angle device, the Drop Shape Analyzer DSSA25, equipped with a video system (Krüss, Hamburg, Germany) and controlled using Krüss Advance software (version 1.3.0.0). The contact angle was measured by dropping 5 µL of ultrapure water on the surface of the membrane through a needle attached to the instrument and determining the mean value of the contact angle over a period of 60 s.

Scanning electron microscopy (SEM) images were acquired using a field emission scanning electron microscope (FE-SEM, S-4100; Hitachi, Tokyo, Japan). Prior to analysis, membrane samples were mounted on aluminium stubs and carbon-coated (K950 turbo evaporator, Emitech, Montigny-le-Bretonneux, France). Image acquisition and processing were performed using Quartz PCI software (Vancouver, Canada) to evaluate membrane morphology, homogeneity, and interfacial adhesion between the polymeric layer and the porous supports.

The thickness of the membranes was measured using a digital micrometer (Mitutoyo Digimatic Micrometer, Model MDC-25MXT, Code 293-234-30, Mitutoyo Corp., Kawasaki, Japan).

Fourier-transform infrared spectroscopy (FT-IR) spectra were recorded using an Agilent Cary 630 FTIR spectrometer (Agilent Technologies, Mulgrave, Australia) equipped with a diamond ATR accessory.

Thermal properties of the membranes were investigated by thermogravimetric analysis using a TGA/DSC instrument (Mettler Toledo, Barcelona, Spain). Measurements were performed on approximately 5 mg of sample over a temperature range of 30–650 °C at a heating rate of 10 °C min^−1^ under a nitrogen atmosphere (40 mL min^−1^). The pristine supports and the corresponding membranes were analysed for comparison.

### 2.4. Mass Loss Measurements

The stability of the membranes was evaluated by monitoring their mass variation after contact with aqueous media. Membrane segments with an approximate area of 4 cm^2^ were cut from different regions of each membrane, including free-standing PIMs, filter paper–PIMs, and Durapore^®^–PIMs, for both CTA and PVC polymers. Each segment was immersed in 50 mL of ultrapure water or 0.1 M NaCl solution and kept under continuous shaking using an orbital mixer for 24 h.

Prior to and after immersion, the membranes were carefully dried and weighed. For supported PIMs, the mass of the pristine support was subtracted from the total mass in order to account only for the possible loss of the PIM phase. The percentage of mass loss was calculated using Equation (1) [31]:

(1) $$\mathrm{Mass}\;\mathrm{Loss}\;\left(\%\right)=\;\frac{W_{\left(0\right)}-W_{\left(f\right)}}{W_{\left(0\right)}}\;\times 100$$

where *W*_(0)_ is the initial membrane weight, and *W*_(*f*)_ is the final membrane weight after 24 h immersed in aqueous solutions. All experiments were carried out at a room temperature of 22 ± 1 °C and were run in triplicate.

### 2.5. As(V) Transport in a Two-Compartment Cell

**Figure 2:**
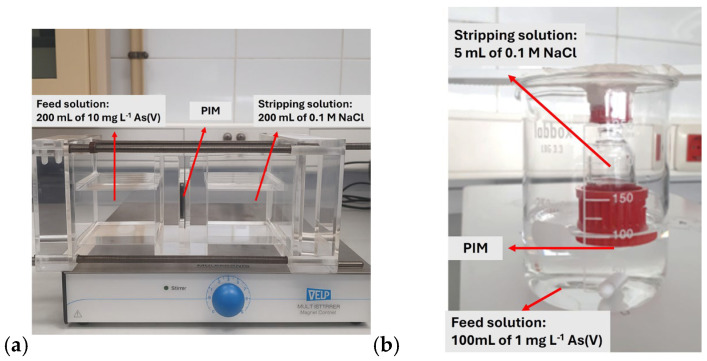
Instrumental setups: (**a**) two-compartment transport cell; (**b**) PIM device.

To evaluate the effect of the supporting substrates on As(V) transport, experiments were performed using a conventional two-compartment transport cell. The setup is shown in Figure 2a. A circular membrane sample (effective area: 12.56 cm^2^) was placed between the feed and receiving compartments. The feed phase consisted of 200 mL of an aqueous solution containing 10 mg L^−1^ As(V), while the receiving phase contained 200 mL of 0.1 M NaCl solution. Both compartments were magnetically stirred to ensure homogeneous conditions on each side of the membrane, and all experiments were conducted at room temperature (22 ± 1 °C). Aliquots were periodically withdrawn from both phases at 1 h intervals over a total transport time of 24 h. Arsenic concentrations were determined by inductively coupled plasma optical emission spectrometry (ICP-OES, Agilent 5100 Vertical Dual 206 View ICP-OES, Agilent Technologies, Tokyo, Japan).

The transport efficiency (*TE*, %) was calculated using Equation (2) [27]:

(2) $$\mathrm{TE}\;\left(\%\right)=\;\frac{{\left[\mathrm{As}\right]}_{s,t}}{{\left[\mathrm{As}\right]}_{f,0}}\;\times 100$$

where [*As*]*_s_*_,*t*_ represents the accumulated arsenic concentration in the stripping phase in time *t* (mg L^−1^) and [*As*]*_f_*_,0_ is the initial concentration of *As* in the feed phase (mg L^−1^).

The experimental data were also expressed in terms of *As*(*V*) permeability, *P* (cm min^−1^), defined in Equation (3) [5]:

(3) $$P\;=\;\frac{-d[\mathrm{As}(V)]}{dt}\frac{V}{A}\frac{1}{\left[\mathrm{As}(V)\right]}$$

where *A* is the effective membrane area (cm^2^) and *V* is the volume of the feed solution (mL).

Integration of Equation (3), assuming that *P* remains constant, gives the following relation:

(4) $$ln\frac{{\left[\mathrm{As}(V)\right]}_{\mathrm{feed}(t)}}{{\left[\mathrm{As}(V)\right]}_{\mathrm{feed}(0)}}\;=-\frac{A}{V}\mathrm{Pt}$$

where [*As*(*V*)]*_feed_*_(0)_ is the initial analyte concentration in the feed phase, while [*As*(*V*)]*_feed_*_(*t*)_ is the analyte concentration remaining in the feed compartment at a certain time. Thus, *P* values can be obtained from the slope of the linear representation ln[*As*(*V*)]*_feed_*_(*t*)_/[*As*(*V*)]*_feed_*_(0)_ versus time.

### 2.6. As(V) Preconcentration and Reusability

The applicability of the supported membranes for As(V) preconcentration and the membrane reusability were investigated using a dedicated membrane-based device previously described elsewhere [24,28] and shown in Figure 2b. In this configuration, the membrane (exposed area: 1.76 cm^2^) separates the feed and receiving phases. The feed phase consisted of 100 mL of an aqueous solution containing 1 mg L^−1^ As(V) and was continuously agitated, while the receiving phase comprised 5 mL of 0.1 M NaCl solution and was kept stagnant. The asymmetric phase volumes enable preconcentration of arsenate in the receiving solution.

Membrane reusability was evaluated by performing consecutive transport cycles, with renewal of both the feed and receiving phases every 24 h. At the end of each cycle, the receiving phase was collected for analysis, and the transport efficiency (*TE*, %) was calculated according to Equation (5) [24]:

(5) $$\mathrm{TE}\;(\%)=\left(\frac{V_{s}}{V_{f}}\right)\;\left(\frac{{\left[\mathrm{As}\right]}_{\mathrm{rec}(t)}}{{\left[\mathrm{As}\right]}_{\mathrm{feed}(0)}}\right)\;\times \;100$$

where [*As*]*_rec_*_(*t*)_ denotes the *As* concentration accumulated in the receiving phase at time *t* (mg L^−1^); [*As*]*_feed_*_(0)_ is the initial *As* concentration in the feed phase (mg L^−1^); and *V_s_* and *V_f_* are the volumes of receiving and feed solutions, respectively.

### 2.7. Statistical Analysis

Statistical analysis of TE values was performed using one-way analysis of variance (ANOVA) to evaluate differences between consecutive transport cycles for each supported membrane. A significance level of *p* < 0.05 was adopted. Results are expressed as mean ± standard deviation of independent experiments (*n* = 3).

## 3. Results and Discussion

### 3.1. Supported PIM Characterization

The newly developed supported PIMs were structurally and chemically characterized by contact angle measurements, SEM, FTIR and TGA analyses.

#### 3.1.1. Contact Angle Measurements

Contact angle measurements were performed to evaluate the surface wettability of the pristine supports, free-standing PIMs, and supported configurations (Table 1).

The pristine Durapore^®^ PVDF membrane exhibited a highly hydrophobic surface (123.3 ± 0.5°), whereas the cellulose filter paper was too hydrophilic to allow reliable contact angle determination. Free-standing CTA- and PVC-based PIMs showed contact angles of 20 ± 3° and 40 ± 2°, respectively, confirming their predominantly hydrophilic character (θ < 90°) [32]. The increase in hydrophilicity upon incorporation of ionic carriers into polymer matrices has been widely reported. In previous studies using Aliquat 336 derivatives such as trioctylmethylammonium thiosalicylate (TOMATS), the addition of 50% carrier significantly reduced the contact angle of both CTA and PVC polymers, demonstrating that ionic extractants enhance membrane polarity and surface wettability [33].

Moreover, the values reported in the literature for CTA-based membranes containing Aliquat 336 typically fall in the range of 15–30°, depending on the carrier content and membrane composition, while PVC-based systems often exhibit contact angles above 35°. Therefore, the values obtained in the present work are in good agreement with previously reported data, confirming the reliability of the membrane preparation and characterization procedures [5].

The lower contact angle observed for CTA-based PIMs compared to PVC-based ones is consistent with the intrinsic physicochemical properties of the two polymers. CTA contains polar acetyl and hydroxyl groups that promote hydrogen bonding with water, while PVC is relatively more hydrophobic. Similar trends have been reported by Vázquez et al., who demonstrated that CTA-based PIMs generally present lower contact angles than PVC-based membranes due to their higher polarity and water affinity [29].

For supported membranes, the surface wettability was clearly influenced by the nature of the substrate. In filter paper–PIMs, the contact angle further decreased for both polymers, indicating a dominant contribution of the hydrophilic cellulose fibres. Conversely, PVC-based Durapore^®^–PIM exhibited a higher contact angle (47 ± 1°) than the corresponding free-standing PVC PIM, suggesting that the hydrophobic PVDF substrate contributes to the overall surface behaviour of the composite membrane. Such interplay between polymer matrix and support has been described in composite membrane systems, where surface wettability reflects both the intrinsic properties of the active layer and the underlying porous structure [34,35,36].

#### 3.1.2. Scanning Electron Microscopy

SEM was employed to investigate both the surface morphology and cross-sectional architecture of the supported PIMs prepared on cellulose filter paper and Durapore^®^ substrates. The analysis aimed to determine the uniformity and integrity of the deposited PIM layer into the porous structure of the supports as mechanical scaffolds for CTA- and PVC-based membranes.

**Figure 3:**
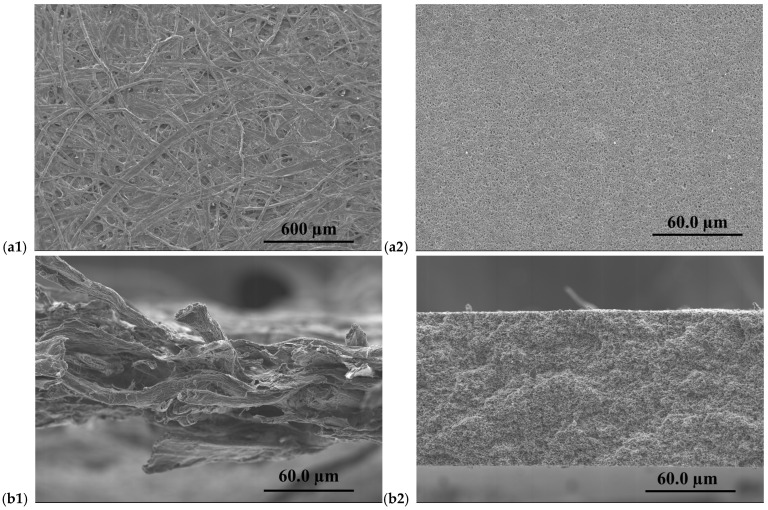
SEM images of surface and cross-section of cellulose filter paper (**a1**,**b1**) and Durapore^®^ membrane (**a2**,**b2**).

As a first step, the pristine support materials were examined by SEM (see Figure 3). The cellulose filter paper exhibits a highly open and heterogeneous fibrous network composed of randomly oriented cellulose fibres (Figure 3(a1)). This structure results in a porous architecture with interconnected voids and a high surface area. The corresponding cross-sectional image (Figure 3(b1)) reveals a multilayered fibrous arrangement with significant thickness and internal voids, which can facilitate the physical entrapment and distribution of the PIM phase within the cellulose matrix.

In contrast, the Durapore^®^ membrane displays a much more uniform and compact surface morphology (Figure 3(a2)), characterized by a fine, homogeneous pore structure. The cross-sectional image (Figure 3(b2)) shows a well-defined and relatively dense membrane architecture with a more consistent thickness. This morphology is typical of Durapore^®^ membranes designed for filtration applications and provides a mechanically stable and chemically inert platform for supporting thin functional layers.

**Figure 4:**
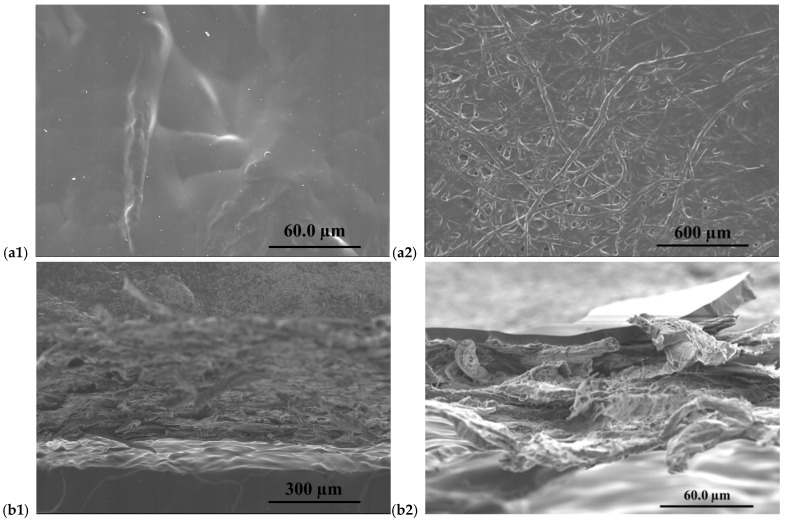
SEM images of surface and cross-section of CTA-based filter paper–PIM (**a1**,**b1**), and PVC-based filter paper–PIM (**a2**,**b2**).

Figure 4 shows SEM observations of the cellulose filter paper-supported PIMs. In this case, they reveal a morphology strongly influenced by the intrinsic fibrous architecture of the filter paper substrate. The use of a low PIM loading results in thin supported membranes, where the underlying cellulose fibres remain partially visible at the surface. This indicates that the PIM phase does not form a thick continuous film but is instead distributed at or near the support surface.

Clear differences are observed between CTA- and PVC-based systems when deposited onto cellulose filter paper. Surface SEM images show that CTA-based membranes (Figure 4(a1)) partially penetrate and spread within the cellulose fibre network. This behaviour is consistent with the higher polarity of CTA and its stronger interaction with the hydrophilic cellulose substrate, leading to a more homogeneous distribution of the PIM phase across the support. In contrast, PVC-based PIMs (Figure 4(a2)) form a more distinct surface layer, with the underlying cellulose fibres remaining clearly visible. This indicates a more superficial deposition of the polymer phase, suggesting limited interaction with the fibrous network and reduced penetration into the substrate.

Cross-sectional SEM images further elucidate these structural differences. For PVC-based PIMs (Figure 4(b2)), a relatively continuous and well-defined layer is observed on top of the cellulose substrate. The interface between the PIM layer and the fibrous support is clearly distinguishable, confirming that the membrane is primarily confined to the outer region of the filter paper. Conversely, CTA-based membranes (Figure 4(b1)) exhibit a less sharply defined interface, with partial infiltration of the polymer phase into the upper layers of the cellulose fibres. This results in a more gradual transition between the membrane and the support, indicating stronger polymer–substrate interactions during solvent casting.

These observations are consistent with the contact angle measurements discussed previously. The lower contact angle of CTA-based PIMs reflects enhanced surface polarity and improved wetting behaviour, which favours penetration into the cellulose structure. In comparison, the higher contact angle measured for PVC-based systems supports their more surface-confined morphology and the clearer visibility of the underlying fibres.

**Figure 5:**
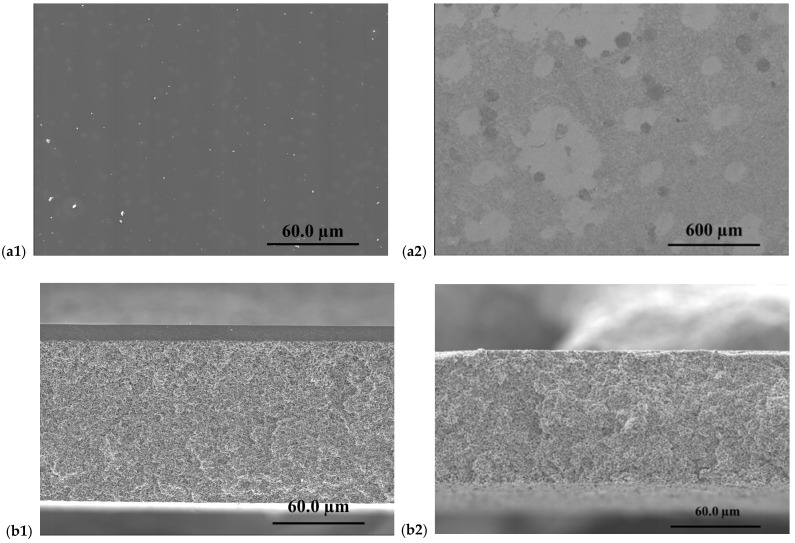
SEM images of surface and cross-section of CTA-based Durapore^®^–PIM (**a1**,**b1**), and PVC-based Durapore-PIM (**a2**,**b2**).

In contrast to the cellulose-supported systems, SEM images of the Durapore^®^–PIMs show a more compact and homogeneous morphology for both CTA- and PVC-based membranes (Figure 5).

Surface images show the formation of a uniform PIM layer covering the porous Durapore^®^ substrate, with limited visibility of the underlying pore structure (Figure 5(a1,a2)). This indicates that the PIM phase spreads predominantly over the surface of the PVDF membrane, with minimal penetration into its pore network.

Cross-sectional images further confirm a well-defined layered configuration, where the PIM phase forms a continuous coating on top of the Durapore^®^ support (Figure 5(b1,b2)). The supported layer appears uniform in thickness across the membrane, suggesting good reproducibility of the casting process. No evidence of pore collapse, delamination, or structural disruption is observed for either polymer, indicating that the Durapore^®^ substrate acts as a mechanically stable and chemically inert platform for PIM immobilization. These observations are in line with the higher hydrophobicity of the Durapore^®^ support, which limits polymer infiltration and promotes surface-confined layer formation.

#### 3.1.3. Fourier-Transform Infrared Spectroscopy

**Figure 6:**
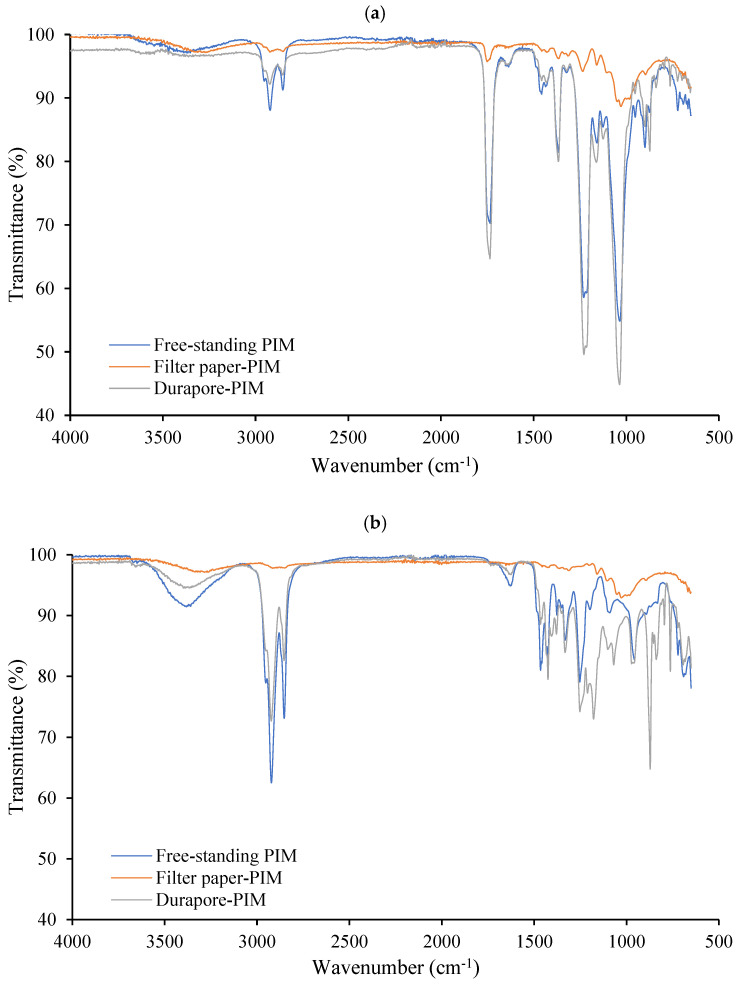
FTIR spectra of (**a**) CTA-based PIMs and (**b**) PVC-based PIMs, including free-standing membranes and membranes supported on cellulose filter paper and Durapore^®^ substrate.

FTIR spectroscopy was used to assess the chemical integrity of PIMs based on CTA and PVC after immobilization on both substrates. Figure 6a shows the spectra corresponding to CTA-based membranes, while Figure 6b presents the spectra of PVC-based systems. In both cases, the spectra of the supported membranes are compared with those of the corresponding free-standing PIMs.

For the CTA-based membranes (Figure 6a), the free-standing PIM exhibits the characteristic absorption bands of the CTA matrix, including the intense carbonyl stretching vibration at approximately 1735 cm^−1^, aliphatic C–H stretching bands in the 2950–2850 cm^−1^ region, and C–O–C stretching vibrations in the 1210–1030 cm^−1^ range. Contributions from the extractant phase (Aliquat 336) are reflected mainly in the aliphatic C–H stretching region and at lower wavenumbers, consistent with the presence of long alkyl chains and a quaternary ammonium salt structure. The spectra of the cellulose- and Durapore^®^-supported CTA-based membranes closely resemble that of the free-standing PIM, with no additional bands or significant shifts observed. A weak broad O–H stretching band around 3300–3500 cm^−1^ can be attributed to the cellulose substrate.

Similarly, the PVC-based membranes (Figure 6b) show the characteristic FTIR features of the PVC matrix, including aliphatic C–H stretching vibrations in the 2950–2850 cm^−1^ region and C–H bending modes in the 1450–1250 cm^−1^ range, together with bands at lower wavenumbers associated with C–C and C–Cl vibrations. As observed for CTA-based systems, the spectra of the supported PVC-based membranes closely match that of the corresponding free-standing PIMs, with no new absorption bands or meaningful band shifts detected upon support on either cellulose filter paper or Durapore^®^.

Overall, the FTIR results demonstrate that, for both CTA- and PVC-based PIMs, immobilization does not induce chemical modification of the membrane components, and, therefore, the supported architectures rely on physical immobilization rather than chemical interaction.

#### 3.1.4. Thermogravimetric Analysis

**Figure 7:**
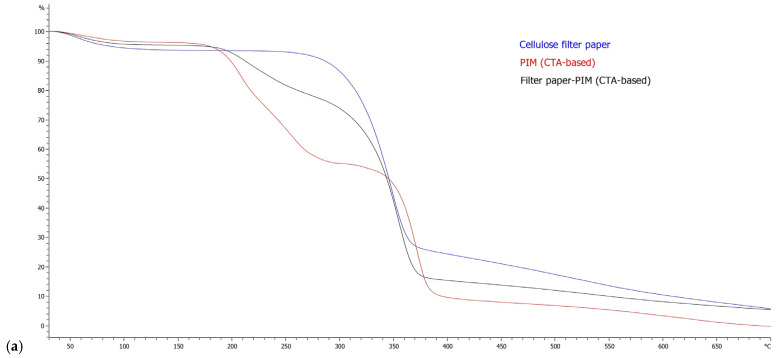

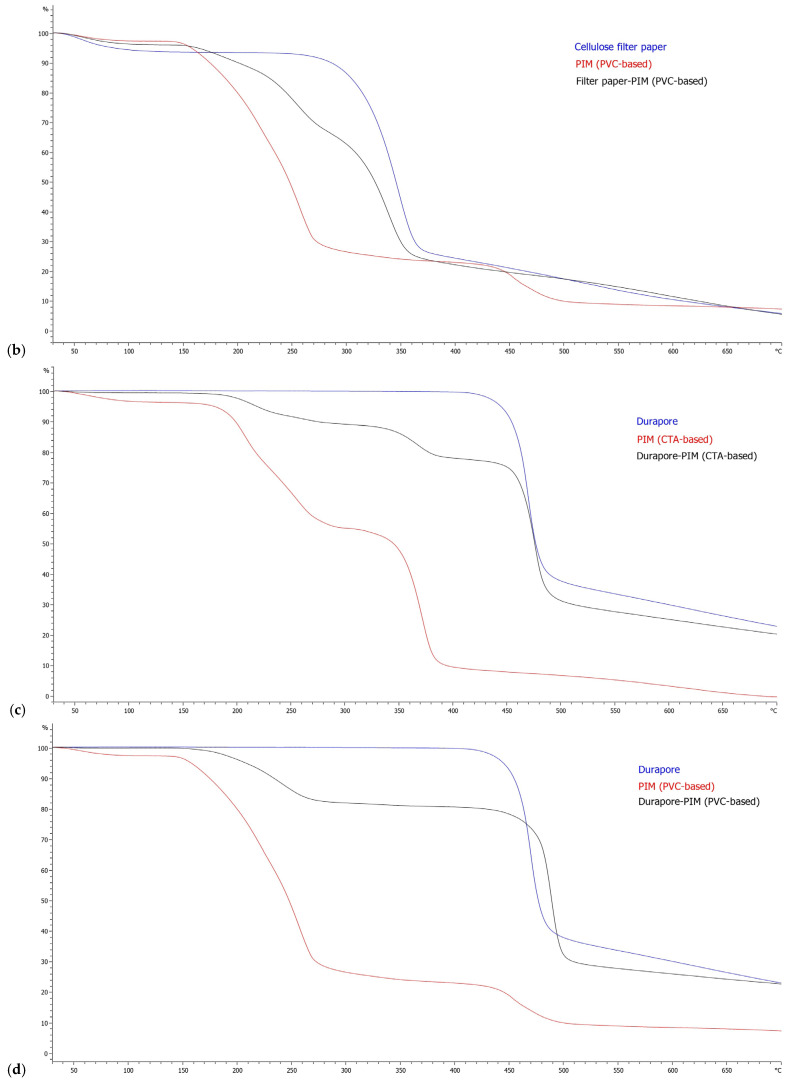
Thermogravimetric profiles of (**a**) pristine cellulose filter paper, free-standing CTA-based PIM, and cellulose-supported CTA-based PIM; (**b**) pristine cellulose filter paper, free-standing PVC-based PIM, and cellulose-supported PVC-based PIM; (**c**) pristine Durapore^®^ membrane, free-standing CTA-based PIM, and Durapore^®^-supported CTA-based PIM; (**d**) pristine Durapore^®^ membrane, free-standing PVC-based PIM, and Durapore^®^-supported PVC-based PIM.

Thermogravimetric analysis was employed to evaluate the thermal behaviour of the supported PIMs and to assess the compatibility between the functional membrane and the porous substrates (see Figure 7a–d).

Figure 7a,b correspond to the TGA of cellulose-supported PIMs. As can be seen, the pristine cellulose filter paper exhibits the characteristic thermal behaviour of cellulosic materials, with a minor mass loss at low temperatures attributed to moisture release, followed by a main degradation step occurring between approximately 300 and 370 °C associated with cellulose backbone decomposition and subsequent char evolution at higher temperatures [37].

When supported on cellulose filter paper, both CTA- and PVC-based PIMs exhibit intermediate thermal behaviour between the pristine support and the corresponding free-standing membranes. The supported membranes display smoother and more gradual mass-loss profiles in the intermediate temperature region, consistent with the reduced loading and physical distribution of the PIM phase within the porous cellulose network. Importantly, no new degradation events are observed for either polymer system, indicating that the cellulose support does not induce chemical modification or destabilization of the PIM components.

Comparing CTA- and PVC-based systems on cellulose, both exhibit similar composite behaviour governed by the superposition of substrate and PIM contributions. However, PVC-based membranes (Figure 7b) tend to exhibit a more pronounced residual mass at higher temperatures, which is consistent with the known thermal decomposition behaviour of PVC-based materials. These differences reflect intrinsic properties of the polymer matrices rather than an effect of the cellulose support.

Figure 7c,d shows the result for Durapore^®^–PIMs. The pristine Durapore^®^ membrane shows high thermal stability, with negligible mass loss up to approximately 430–450 °C, followed by a sharp degradation step characteristic of Durapore^®^ decomposition [38]. This confirms its suitability as a thermally robust support material.

In the Durapore^®^-supported configurations, both polymer systems display a clear two-domain thermal profile: an initial mass-loss region associated with the PIM phase at lower temperatures, followed by a distinct high-temperature degradation step corresponding to the Durapore^®^ support.

The preservation of the Durapore^®^ degradation event and the absence of additional thermal features indicate that the Durapore^®^ membrane remains chemically inert in the presence of the PIM layer. Furthermore, the PIM-related degradation occurs in a similar temperature range to that observed for the free-standing membranes, confirming that the support does not chemically stabilize or destabilize the functional components.

As observed for cellulose-supported membranes, the differences between CTA- and PVC-based systems supported on Durapore^®^ are mainly reflected in the shape of the PIM-related mass-loss region and the residual mass at higher temperatures

#### 3.1.5. Evaluation of Membrane Stability by Mass Loss Measurements

The stability of PIMs is commonly assessed through mass loss measurements after contact with aqueous media, which provide a practical and widely used indicator of membrane component leaching. Although this method does not directly determine the identity of the released species, previous studies have shown that for PIMs containing ionic liquid extractants such as Aliquat 336, a significant fraction of the observed mass loss can be attributed to extractant leaching into the aqueous phase [29,39].

In this study, the mass loss of supported and free-standing CTA- and PVC-based membranes was evaluated after 24 h of contact with ultrapure water and 0.1 M NaCl, and the results are summarized in Table 2.

Overall, mass losses were significantly higher in ultrapure water than in saline solution for all PIM compositions. This behaviour is fully consistent with previous stability investigations of Aliquat 336-based PIMs. Kagaya et al. [40] demonstrated that PVC-based PIMs containing Aliquat 336 undergo significant mass loss when immersed in deionized water, whereas the presence of dissolved salts markedly suppresses this loss. Similarly, Dahdah et al. [31] reported that the stability of CTA-based PIMs strongly depends on the aqueous medium composition, with significantly reduced mass losses (<5 wt%) observed in 1 M NaNO_3_ and acidic nitrate media. Furthermore, Xu et al. [41] quantified the intrinsic solubility of Aliquat 336 in aqueous media, reporting measurable extractant solubility (≈0.1 g per 100 mL in 2 M HCl), thereby confirming that extractant leakage is an inherent physicochemical phenomenon rather than solely a mechanical instability. Taken together, these studies support the interpretation that the mass losses measured in the present work—particularly under low ionic strength conditions—are predominantly associated with partial carrier solubilization, while the reduced losses observed in 0.1 M NaCl are consistent with the stabilizing effect of ionic strength reported in the literature.

The influence of porous supports on membrane mass loss was assessed by comparing free-standing and supported configurations prepared with identical polymer loadings.

For CTA-based membranes immersed in ultrapure water, supporting the PIM on cellulose filter paper led to a noticeable reduction in mass loss, whereas Durapore^®^-supported membranes showed a more moderate effect. This observation suggests that the cellulose support may provide a degree of physical retention for membrane components under these conditions. A similar trend was observed for PVC-based membranes in ultrapure water. Membranes supported in filter paper exhibited lower mass loss compared to free-standing PIMs, whereas no systematic improvement was observed for Durapore^®^-supported membranes.

However, under NaCl conditions, the mass loss of both CTA- and PVC-based supported PIMs decreased substantially, with differences between free-standing and supported configurations becoming less pronounced.

#### 3.1.6. As(V) Transport in a Two-Compartment Cell

The transport performance of the membranes was first evaluated in a conventional two-compartment cell in order to assess their intrinsic ability to transport As(V) under well-defined conditions.

**Figure 8:**
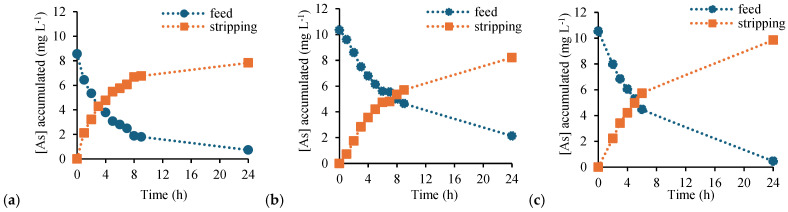
Transient concentration profiles of As(V) in the feed and stripping phases using CTA-based PIMs. (**a**) Free-standing PIM, (**b**) filter paper–PIM, and (**c**) Durapore^®^–PIM. Feed solution: 200 mL of As(V) 10 mg L^−1^. Stripping solution: 200 mL of 0.1 M NaCl. PIM total mass: 0.2 g.

Figure 8 shows the transport profiles of As(V) through CTA-based membranes, including the free-standing PIM (a) and the supported configurations prepared on cellulose filter paper (b) and Durapore^®^ membrane (c). In all cases, effective transfer of As(V) from the feed to the stripping phase was observed, confirming that membrane functionality was preserved despite the reduced polymer loading and the incorporation of porous supports.

Although comparable final TE were achieved after 24 h, differences in transport kinetics were clearly observed. The free-standing CTA-based PIM exhibited the fastest As(V) transfer, whereas both supported membranes required longer times to approach equilibrium. This behaviour is reflected in the calculated *P* coefficients, which decreased from 0.046 cm·min^−1^ for the free-standing membrane to 0.024 cm·min^−1^ and 0.036 cm·min^−1^ for the filter paper–PIM and Durapore^®^–PIM, respectively. The lower *P* can be attributed to the additional diffusion resistance introduced by the porous supports, which increases the effective transport path length and may impose partial mass transfer limitations within the support structure.

It is worth noting that in a previous study by our group, CTA/Aliquat 336 PIMs prepared with higher membrane loadings (total membrane mass = 0.4 g) exhibited permeability values as high as 0.26 cm·min^−1^ [27]. The reduced *P* values observed in the present work are therefore consistent with the significantly lower total membrane mass employed. Importantly, despite this decrease in permeability, the final TE after 24 h remained comparable, indicating that reducing the amount of membrane-forming reagents primarily affects transport kinetics rather than overall extraction performance.

**Figure 9:**
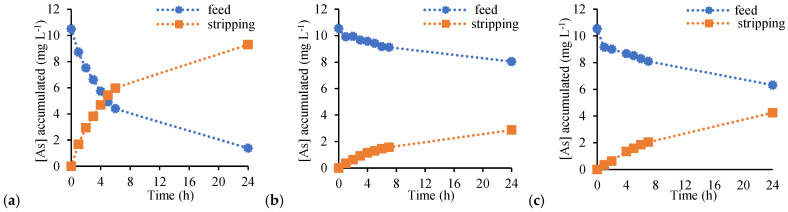
Transient concentration profiles of As(V) in the feed and stripping phases using PVC-based PIMs. (**a**) Free-standing PIM, (**b**) filter paper–PIM, and (**c**) Durapore^®^–PIM. Feed solution: 200 mL of As(V) 10 mg L^−1^. Stripping solution: 200 mL of 0.1 M NaCl. PIM total mass: 0.2 g.

However, the transport behavior of PVC-based membranes (Figure 9) exhibited a pronounced dependence on the total membrane mass. At low loading (total PIM mass of 0.2 g), the free-standing PVC-based PIM showed relatively high TE = 88.6% and *P* = 0.038 cm·min^−1^. In contrast, the supported configurations performed significantly worse under the same conditions, with TE values of 27.2% and 40.3% for filter paper–PIM and Durapore^®^–PIM, respectively, and *P* below 0.005 cm·min^−1^. These results indicate that, at low membrane mass, the supported PVC-based membranes are unable to sustain efficient transport within the experimental time frame.

**Figure 10:**
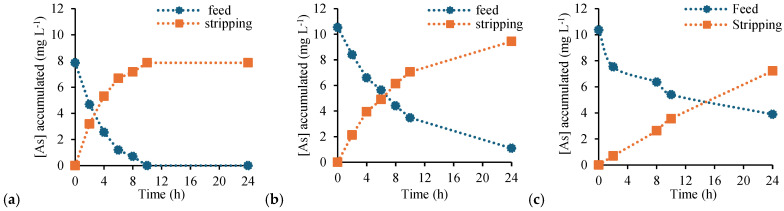
Transient concentration profiles of As(V) in the feed and stripping phases using PVC-based PIMs. (**a**) Free-standing PIM, (**b**) filter paper–PIM, and (**c**) Durapore^®^–PIM. Feed solution: 200 mL of As(V) 10 mg L^−1^. Stripping solution: 200 mL of 0.1 M NaCl. PIM total mass: 0.8 g.

Increasing the total membrane mass to 0.8 g, corresponding to the typical formulation employing 0.4 g of PVC and 0.4 g of extractant (50 wt% polymer/50 wt% carrier), as commonly reported in the literature [24,29], resulted in a marked improvement in transport performance for all PVC-based membranes (Figure 10). The free-standing PIM reached complete As(V) transport within 10 h with a *P* of 0.082 cm·min^−1^. Importantly, the supported membranes also showed a substantial enhancement in both TE and *P*: the filter paper–PIM reached a TE of 89.6% with a *P* of 0.028 cm·min^−1^, while the Durapore^®^–PIM exhibited a TE of 69.7% (24 h) and a *P* of 0.011 cm·min^−1^. These increases confirm that insufficient membrane loading is the main factor limiting transport performance in supported PVC-based systems.

The stronger sensitivity of PVC-based membranes to membrane loading can be rationalized by considering the structural features of the polymer matrix. SEM analysis revealed that PVC-based PIMs tend to form more compact and well-defined surface layers, particularly when supported on porous substrates. At low total mass, this compact morphology may result in an insufficiently continuous or accessible carrier-rich phase, leading to severely reduced *P*. Increasing the membrane mass enhances the continuity and effective thickness of the functional phase, thereby restoring transport efficiency.

Overall, the superior performance of CTA at low loading can be attributed to the combined effects of (i) higher polarity and hydrophilicity, (ii) better wetting and interaction with hydrophilic supports, and (iii) possible formation of more continuous and accessible transport pathways under reduced membrane thickness [5,14,29]. In contrast, PVC-based PIMs require a higher polymer and carrier loading to achieve efficient transport in supported configurations.

#### 3.1.7. As(V) Preconcentration and Reusability

To evaluate the applicability of the supported PIMs for As(V) preconcentration and to assess their reusability, transport experiments were performed using a dedicated membrane-based device that enables the preconcentration of As(V) in the receiving solution, while repeated transport cycles allow evaluation of membrane performance over time.

**Figure 11:**
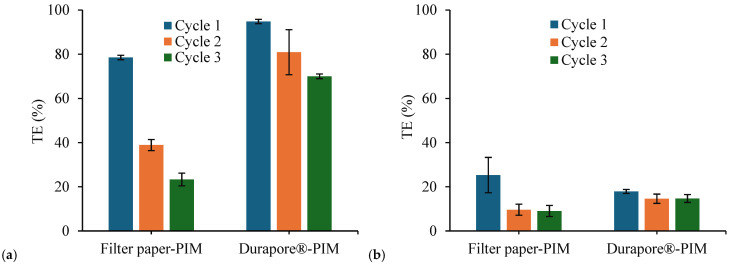
Transport efficiency of CTA-based supported PIMs (**a**) and PVC-based PIMs (**b**) over three consecutive transport cycles. Experimental conditions: feed solution—100 mL of 1 mg L^−1^ As(V); receiving phase—5 mL of 0.1 M NaCl; contact time—24 h per cycle.

Figure 11 shows the TE % of As(V) over three consecutive transport cycles for each membrane. A similar number of reuse cycles has been reported in recent membrane studies for arsenic removal, as reported in [42].

In the case of CTA-based supported membranes (Figure 11a), both filter paper–PIM and Durapore^®^–PIM enabled effective As(V) preconcentration during the first cycle, confirming their suitability for operation in the device configuration. However, distinct behaviors were observed upon repeated use.

For the filter paper–PIM, TE decreased progressively from the first to the third cycle. Statistical analysis (one-way ANOVA) confirmed that these differences between cycles were highly significant (*p* < 0.001), indicating a pronounced loss of transport performance upon reuse.

In contrast, the Durapore^®^–PIM exhibited high initial performance and retained comparatively higher TE values over successive cycles. Although a statistically significant decrease between cycles was also observed for this system (*p* < 0.01), the magnitude of the decline was markedly less pronounced than that observed for the cellulose-supported membrane. After the third cycle, Durapore^®^–PIM still maintained substantially higher TE values, evidencing improved operational stability.

The different reusability trends can be attributed to the nature of the supporting substrates and the resulting membrane architecture. In the cellulose-based support, the hydrophilic and highly porous fibre network may promote redistribution or gradual loss of the carrier-rich phase during repeated contact with aqueous media, leading to a progressive reduction in effective transport sites. Conversely, the hydrophobic Durapore^®^ support likely favors the formation of a more confined and continuous functional layer, limiting carrier migration and mitigating performance decay over repeated cycles.

These results demonstrate that, while both supports enable efficient initial transport, the long-term functional stability of CTA-based supported PIMs is strongly influenced by the ability of the substrate to retain and stabilize the active membrane phase.

For PVC-based supported membranes prepared at low total mass (Figure 11b), TE values were already low in the first cycle and remained within a similar range over subsequent cycles. For filter paper–PIM, statistical analysis revealed significant differences between cycles (*p* < 0.05), mainly associated with the drop observed after the first cycle. However, no statistically significant differences were detected between cycles for the PVC-based Durapore^®^–PIM (*p* > 0.05).

In contrast to CTA-based membranes, the absence of a pronounced progressive decline in TE for PVC-based systems suggests that membrane degradation is not the dominant factor affecting performance. Instead, the consistently low efficiencies indicate that transport is primarily constrained by intrinsic limitations of the PVC-based membrane matrix and its architecture, rather than by support-dependent destabilization effects.

## 4. Conclusions

This study demonstrates, for the first time, the feasibility of preparing PIMs supported on porous substrates as a strategy to significantly reduce membrane material usage while maintaining functional As(V) transport. CTA- and PVC-based PIMs containing Aliquat 336 were successfully deposited onto cellulose filter paper and Durapore^®^ supports using a total membrane mass well below that commonly reported for conventional PIMs.

The results reveal that membrane performance is strongly influenced by the polymer matrix, support nature, and operating conditions. CTA-based supported membranes exhibited transport efficiencies comparable to free-standing PIMs, whereas PVC-based systems showed a stronger dependence on membrane loading. Preconcentration and reusability studies further highlighted distinct roles of the supports: cellulose-based membranes performed well in initial transport, making them suitable for single-use or short-term applications, while Durapore^®^-supported membranes showed improved retention of transport efficiency over repeated cycles.

Overall, these findings establish supported PIMs as a promising and adaptable platform for reagent-efficient membrane-based separations and provide practical guidelines for selecting membrane composition and support according to the intended application.

## Figures and Tables

**Table 1:** Contact angle values (°) of pristine supports, free-standing PIMs, and supported PIM based on CTA and PVC polymers.

Support/Membrane	Contact Angle (Average ± SD) (°)	
	CTA	PVC
Filter paper	Not measurable	
Durapore^®^	123.3 ± 0.5	
Free-standing PIMs	20 ± 3	40 ± 2
Filter paper–PIM	10 ± 1	22 ± 4
Durapore^®^–PIM	nd	47 ± 1

Nd. Not determined.

**Table 2:** Mass loss (%) of supported and free-standing PIMs after 24 h contact with aqueous media.

Polymer	Aliquat 336	Medium	Free-Standing PIM	Filter Paper–PIM	Durapore^®^–PIM
CTA (0.1 g)	0.1 g	H_2_O	43 ± 2	34 ± 3	38 ± 5
		0.1 M NaCl	14 ± 6	18 ± 1	17 ± 2
PVC (0.1 g)	0.1 g	H_2_O	34 ± 0.2	24 ± 2	38 ± 4
		0.1 M NaCl	17 ± 4	6 ± 1	11 ± 0.3

## Data Availability

The original contributions presented in this study are included in the article. Further inquiries can be directed to the corresponding author.

## References

[1] Kaczorowska M.A. (2022). The Use of Polymer Inclusion Membranes for the Removal of Metal Ions from Aqueous Solutions—The Latest Achievements and Potential Industrial Applications: A Review. Membranes.

[2] Keskin B., Zeytuncu-Gökoğlu B., Koyuncu I. (2021). Polymer inclusion membrane applications for transport of metal ions: A critical review. Chemosphere.

[3] Zhao S., Samadi A., Wang Z., Pringle J.M., Zhang Y., Kolev S.D. (2024). Ionic liquid-based polymer inclusion membranes for metal ions extraction and recovery: Fundamentals, considerations, and prospects. Chem. Eng. J..

[4] Nowik-Zając A., Sabadash V. (2025). Recent Developments in Polymer Inclusion Membranes: Advances in Selectivity, Structural Integrity, Environmental Applications and Sustainable Fabrication. Membranes.

[5] Sellami F., Kebiche-Senhadji O., Marais S., Couvrat N., Fatyeyeva K. (2019). Polymer inclusion membranes based on CTA/PBAT blend containing Aliquat 336 as extractant for removal of Cr(VI): Efficiency, stability and selectivity. React. Funct. Polym..

[6] Bahrami S., Dolatyari L., Shayani-Jam H., Yaftian M.R., Kolev S.D. (2022). On the Potential of a Poly(vinylidenefluoride-co-hexafluoropropylene) Polymer Inclusion Membrane Containing Aliquat^®^ 336 and Dibutyl Phthalate for V(V) Extraction from Sulfate Solutions. Membranes.

[7] Rahman M.M., Islam S., Mubasshira, Islam M.S., Ahammad R., Islam M.A., Hasib M.A., Rahman M.S., Moshwan R., Ehsan M.M. (2026). Polymer Composites in Additive Manufacturing: Current Technologies, Applications, and Emerging Trends. Polymers.

[8] Shahid M.U., Najam T., Islam M., Hassan A.M., Assiri M.A., Rauf A., Rehman A.U., Shah S.S.A., Nazir M.A. (2024). Engineering of metal organic framework (MOF) membrane for waste water treatment: Synthesis, applications and future challenges. J. Water Process Eng..

[9] González-Bermúdez M., López-Lorente Á.I., Lucena R., Cárdenas S. (2023). Paper-based sorptive phases for a sustainable sample preparation. Adv. Sample Prep..

[10] Chaipet T., Fresco-Cala B., Thammakhet-Buranachai C., Lucena R., Cárdenas S. (2025). Sustainable hybrid sorbent based on a thin film gelatin coating over cellulose paper for the determination of steroid hormones in urine and environmental water samples. Microchem. J..

[11] Chaiendoo K., Tuntulani T., Ngeontae W. (2017). A paper-based ferrous ion sensor fabricated from an ion exchange polymeric membrane coated on a silver nanocluster-impregnated filter paper. Mater. Chem. Phys..

[12] Jayawardane B.M., Coo L.D.L.C., Cattrall R.W., Kolev S.D. (2013). The use of a polymer inclusion membrane in a paper-based sensor for the selective determination of Cu(II). Anal. Chim. Acta.

[13] Wu Y.L., Xu S., Wang T., Wang C.F. (2018). Enhanced Metal Ion Rejection by a Low-Pressure Microfiltration System Using Cellulose Filter Papers Modified with Citric Acid. ACS Appl. Mater. Interfaces.

[14] Nitti F., Selan O.T.E., Hoque B., Tambaru D., Djunaidi M.C. (2022). Improving the Performance of Polymer Inclusion Membranes in Separation Process Using Alternative Base Polymers: A Review. Indones. J. Chem..

[15] Mahdavi H., Zeinalipour N., Kerachian M.A., Heidari A.A. (2022). Preparation of high-performance PVDF mixed matrix membranes incorporated with PVDF-g-PMMA copolymer and GO@SiO_2_ nanoparticles for dye rejection applications. J. Water Process Eng..

[16] O’Bryan Y., Cattrall R.W., Truong Y.B., Kyratzis I.L., Kolev S.D. (2016). The use of poly(vinylidenefluoride-co-hexafluoropropylene) for the preparation of polymer inclusion membranes. Application to the extraction of thiocyanate. J. Memb. Sci..

[17] Wang B., Wang Y., Xu T. (2023). Recent Advances in the Selective Transport and Recovery of Metal Ions using Polymer Inclusion Membranes. Adv. Mater. Technol..

[18] Chen L., Chen J. (2016). Asymmetric Membrane Containing Ionic Liquid [A336][P507] for the Preconcentration and Separation of Heavy Rare Earth Lutetium. ACS Sustain. Chem. Eng..

[19] Wang Z., Sun Y., Tang N., Miao C., Wang Y., Tang L., Wang S., Yang X. (2019). Simultaneous extraction and recovery of gold(I) from alkaline solutions using an environmentally benign polymer inclusion membrane with ionic liquid as the carrier. Sep. Purif. Technol..

[20] Sellami F., Kebiche-Senhadji O., Marais S., Colasse L., Fatyeyeva K. (2020). Enhanced removal of Cr(VI) by polymer inclusion membrane based on poly(vinylidene fluoride) and Aliquat 336. Sep. Purif. Technol..

[21] Kazemi D., Yaftian M.R. (2024). PVDF-HFP-based polymer inclusion membrane functionalized with D2EHPA for the selective extraction of bismuth(III) from sulfate media. Sci. Rep..

[22] Kang G., Cao Y. (2014). Application and modification of poly(vinylidene fluoride) (PVDF) membranes—A review. J. Memb. Sci..

[23] Ali N., Azeem S., Khan A., Khan H., Kamal T., Asiri A.M. (2020). Experimental studies on removal of arsenites from industrial effluents using tridodecylamine supported liquid membrane. Environ. Sci. Pollut. Res..

[24] Vera R., Anticó E., Fontàs C. (2018). The use of a polymer inclusion membrane for arsenate determination in groundwater. Water.

[25] Govindappa H., Bhat M.P., Uthappa U.T., Sriram G., Altalhi T., Kumar S.P., Kurkuri M. (2022). Fabrication of a novel polymer inclusion membrane from recycled polyvinyl chloride for the real-time extraction of arsenic (V) from water samples in a continuous process. Chem. Eng. Res. Des..

[26] Zawierucha I., Nowik-Zajac A., Malina G. (2020). Selective removal of As(V) ions from acid mine drainage using polymer inclusion membranes. Minerals.

[27] Güell R., Anticó E., Kolev S.D., Benavente J., Salvadó V., Fontàs C. (2011). Development and characterization of polymer inclusion membranes for the separation and speciation of inorganic As species. J. Memb. Sci..

[28] Fontàs C., Vera R., Batalla A., Kolev S.D., Anticó E. (2014). A novel low-cost detection method for screening of arsenic in groundwater. Environ. Sci. Pollut. Res..

[29] Vázquez M.I., Romero V., Fontàs C., Anticó E., Benavente J. (2014). Polymer inclusion membranes (PIMs) with the ionic liquid (IL) Aliquat 336 as extractant: Effect of base polymer and IL concentration on their physical-chemical and elastic characteristics. J. Memb. Sci..

[30] Govindappa H., Abdi G., Uthappa U.T., Sriram G., Han S.S., Kurkuri M. (2023). Efficient separation of arsenic species of oxyanion As (III) and As (V) by using effective polymer inclusion membranes (PIM). Chemosphere.

[31] Dahdah H., Sellami F., Dekkouche S., Benamor M., Senhadji-Kebiche O. (2023). Stability study of polymer inclusion membranes (PIMs) based on acidic (D2EHPA), basic (Aliquat 336) and neutral (TOPO) carriers: Effect of membrane composition and aqueous solution. Polym. Bull..

[32] Soo J.A.L., Shoparwe N.F., Otitoju T.A., Mohamad M., Tan L.S., Li S., Makhtar M.M.Z. (2021). Characterization and kinetic studies of poly(Vinylidene fluoride-co-hexafluoropropylene) polymer inclusion membrane for the malachite green extraction. Membranes.

[33] Alcalde B., Elias G., Kolev S.D., Méndez J.A., Díez S., Oliver-Ortega H., Anticó E., Fontàs C. (2024). A Comprehensive Study on the Effect of Plasticizers on the Characteristics of Polymer Inclusion Membranes (PIMs): Exploring Butyl Stearate as a Promising Alternative. Membranes.

[34] Abdul-Hussein S.T., Alsalhy Q.F., Al-Furaiji M.H., Russo F., Chiappetta G., Di Luca G., Figoli A. (2024). Systematic investigation of MAX phase (Ti3AlC2) modified polyethersulfone membrane performance for forward osmosis applications in desalination. Arab. J. Chem..

[35] Sadidi M., Hajilary N., Abbasi F. (2023). Fabrication of a new composite membrane consisting of MXene/PES /PEI for biofuel dehydration via pervaporation. Results Eng..

[36] You H.-Y., Yin H.-Y., Zhao J.-H., Xiang Z.-Y. (2025). Preparation of Polyaniline Modified Cellulose Filter Paper and Its Application in Detecting 23 Per- and Polyfluoroalkyl Substances. Sep. Sci..

[37] Calahorra M.E., Cortázar M., Eguiazábal J.I., Guzmán G.M. (1989). Thermogravimetric analysis of cellulose: Effect of the molecular weight on thermal decomposition. Appl. Polym..

[38] Jaleh B., Gavar N., Fakhri P., Muensit N., Taheri S.M. (2015). Characteristics of PVDF membranes irradiated by electron beam. Membranes.

[39] Velikova K., Dudev T., Sarafska T., Kukoc-Modun L., Kolev S.D., Spassov T. (2025). Assessing the Stability of Polymer Inclusion Membranes: The Case of Aliquat 336-Based Membranes. Membranes.

[40] Kagaya S., Ryokan Y., Cattrall R.W., Kolev S.D. (2012). Stability studies of poly(vinyl chloride)-based polymer inclusion membranes containing Aliquat 336 as a carrier. Sep. Purif. Technol..

[41] Xu J., Paimin R., Shen W., Wang X. (2003). An Investigation of Solubility of Aliquat 336 in Different Extracted Solutions. Fibers Polym..

[42] Galiano F., Mancuso R., Guazzelli L., Pomelli C.S., Bundschuh J., Rinklebe J., Wang S.L., Apollaro C., Palumbo F., Chiappe C. (2024). Arsenic water decontamination by a bioinspired As-sequestering porous membrane. Nat. Water.

